# Stigma in the context of schools: analysis of the phenomenon of stigma in a population of university students

**DOI:** 10.1186/s12888-016-0734-8

**Published:** 2016-02-09

**Authors:** Luca Pingani, Sara Catellani, Valeria Del Vecchio, Gaia Sampogna, Sarah E. Ellefson, Marco Rigatelli, Andrea Fiorillo, Sara Evans-Lacko, Patrick W. Corrigan

**Affiliations:** Human Resource Development, Local Health Agency of Reggio Emilia, Via Amendola 2, Reggio Emilia, Italy; International PhD School in Clinical and Experimental Medicine, University of Modena and Reggio Emilia, Via del Pozzo 71, Modena, Italy; Department of Psychiatry, University of Naples SUN, Largo Madonna delle Grazie, Naples, Italy; Institute of Psychology, Illinois Institute of Technology, 3424 S. State Street, Chicago, USA; Department of Diagnostic Medicine, Clinical and Public Health, University of Modena and Reggio Emilia, Via del Pozzo 71, Modena, Italy; Institute of Psychiatry, Psychology & Neuroscience, King’s College London, De Crespigny Park, London, SE58AF UK

**Keywords:** Mental health, Psychometrics, Reliability and validity, Stigma, Stereotypes, Questionnaires, University students

## Abstract

**Background:**

Students have stereotyped views about people with mental illness. In particular, they believe that these persons are incurable, dangerous, unpredictable and responsible for their condition. This study aims to investigate the levels of public stigma in an Italian university population.

**Methods:**

The Attribution Questionnaire 27 - Italian Version (AQ-27-I) was administered to a sample of students from the Faculty of Medicine and Surgery of the University of Modena and Reggio Emilia. After examining the psychometric characteristics of the AQ-27-I (Cronbach’s Alpha and Confirmatory Factor Analysis), multiple linear regression analyses were carried out to identify the predictors of stigmatizing attitudes in this population.

**Results:**

Three hundred and eleven students completed the questionnaire, with a response rate of 32.81 % (out of the 948 contacted by email). The AQ-27-I showed good psychometric properties with an α = .68, and the fit indices of the models that partially supported the factor structure and paths. The two variables identified as possible predictors of stigmatizing attitudes (total score of AQ-27-I) were age and time spent reading newspapers.

**Conclusions:**

Antistigma campaigns are needed in university contexts, targeted in particular to students in health professions.

## Background

Stigmatization is a social phenomenon leading to the marginalization of a specific member or a group of the community. Stigma leads to discrimination and loss of dignity as a result of prejudices by other members of the society [1]. Erving Goffman originally defined stigma as a mark or attribute that makes the person “from a whole and usual person to a tainted, discounted one” [2].

Six different types of stigma related to mental illness have been described in the literature: public stigma [3]; structural stigma [4]; self stigma [5]; felt or perceived stigma [6]; experienced stigma [6] and label avoidance [7, 8].

The stigmatized individual is assigned an attribute that makes him/her different and usually less desirable than others. The person is thus downgraded from being a full individual to a discredited person. As a result, the stigmatized person is isolated and marginalized. Stigma against persons with mental illness remains the strongest negative connotation of all social relations [9].

The attribution theory is an example of a social cognitive model that can be used to better understand stigma and in particular the belief that persons with mental illness are responsible for their mental health disorder (“public stigma”). Specifically, Weiner’s attribution theory involves an interest in person’s perceptions of causes of events [10]. Causal beliefs lead to assumptions regarding personal responsibility, which directly impacts a person’s affect (usually in the form of anger or pity). These thoughts of personal responsibility and feelings regulate social behaviors toward the others [10]. If a person is perceived as responsible for his or her mental illness, then people may be angry towards the person, and will marginalize him or her through segregation and coercion. If the person is perceived as not responsible for his or her mental illness, then feelings of pity will emerge. A second concept of public stigma pertains to the notion that persons with mental illness are dangerous. Previously described as “danger appraisal”, those who think that persons with mental illness are dangerous are more likely to react with fears and to avoid them [11, 12]. Discrimination against people with mental illness can result in negative social and health outcomes, such as difficulties in finding a job, discontinuation of studies, decrease in social network, reduced access to mental health care and low adherence to treatment.

Recent studies have reported that medical students believe that persons with mental illness are unpredictable, dangerous, and incurable [13]. Students in social work also endorse a desire to maintain social distance from people with mental illness [14]. Medical and nursing undergraduate students report not having enough information about mental disorders [15]. Previous studies have found that students’ stigmatizing attitudes are influenced by socio-demographic characteristics, such as gender, age and culture, and by a direct or indirect experience of mental illness [13, 14, 16–20].

Over the last thirty years, several efforts have been made in Italy to improve the public understanding and acceptance of persons with mental disorders. In particular, in 1978 the “Basaglia law”, which gradually closed psychiatric hospitals shifting mental health care in the community, should have increased the general public understanding and social contact with persons with serious mental illness [21, 22].

Another aspect we considered in our study is the knowledge of mental illness through newspapers and educational conferences on stigma. Mass media often portray persons with mental illness as dangerous and violent [23], and Italian newspapers often use psychiatric terms, such as schizophrenia, to describe incoherent, dangerous, aggressive, or odd behaviors [24, 25]. Thus, reading newspapers may result in an increase of stigma among the general population, as shown in a previous Italian multicenter study, which compared the opinions about mental illness of the general population, relatives and mental health professionals [26].

In this study, our primary aim was to validate the Italian version of the AQ 27 (AQ-27-I) in a medical student population. In secondary analyses, we assessed and verify possible predictors of stigmatization in the same population. To the best of our knowledge, no study has been carried out in Italy to explore the presence of stigmatizing attitudes in medical students.

## Methods

### Sample

All students (*N* = 948) attending a three-year first level degree course at the Faculty of Medicine and Surgery of the University of Modena and Reggio Emilia during the years 2011 and 2012 received an e-mail invitation to participate in the study, together with the study protocol. A sample of five times the number of questionnaire items is considered the minimum for confirmatory factor analysis: as the questionnaire is composed by 27 items, we needed a minimum of 135 completed questionnaires [27].

After providing informed consent, students who agreed to participate received an online version of the AQ-27-I with an ad-hoc schedule on their socio-demographic characteristics and three additional questions on the knowledge of Italian mental health care organization (i.e., knowledge of the thirtieth anniversary of the Basaglia law), previous educational experiences related to stigma (i.e., participation in scientific conferences) and exposure to information provided by mass media on mental health (i.e., time spent reading newspapers every day). The study obtained the ethical approval by the Board of Presidents of the Educational Courses of the Faculty of Medicine and Surgery of the University of Modena and Reggio Emilia (Italy).

### Instrument description

The AQ-27-I is a 27-item self-administered questionnaire already validated in the Italian and Spanish languages for use with the general population [28, 29]. Respondents are asked to rate their level of agreement with each statement about “Harry”, a 30-year-old single man with schizophrenia, on a Likert scale from 1 (“not at all”) to 9 (“very much”). The AQ-27-I includes 9 subscales, each assessing a typical stereotype about people with mental illness: responsibility, pity, anger, dangerousness, fear, help, coercion, segregation and avoidance. Six items (7, 8, 16, 20, 21, 26) are reverse scored. Globally, the AQ-27-I provides a measure of public stigma. Higher scores indicate greater stigmatization toward Harry. The psychometric properties of the original questionnaire (AQ-27) were examined by two previous confirmatory factor analyses and found the AQ-27 to be acceptable and stable [1, 30]. The AQ-27-I demonstrated an acceptable internal consistency, with a Cronbach’s alpha of 0.82 for the total scale, and a satisfactory test–retest reliability, with intraclass correlation coefficient of 0.72 [28].

### Validation process

Confirmatory factor analysis was performed to assess whether the original theoretical construct could be applied to a population of university students. Model fit was assessed using the following indices: *χ*2, Goodness of Fit Index (GFI > 0.90), Root Mean Square Error of Approximation (RMSEA < 0.05), Adjusted Goodness of Fit Index (AGFI > 0.90) and Comparative Fit Index (CFI > 0.90). Cronbach’s alpha was used to assess instrument reliability (α ≥ 0.65).

### Predictors of stigmatizing attitudes in the university population

The relationship between stigma and (1) age, (2) sex, (3) knowledge of the “Basaglia law”, (4) attendance of a conference on stigma, (5) time spent reading newspapers was explored using a multivariable linear regression for total score and every single subscales of AQ-27-I.

## Results

### Socio-demographic characteristics of the sample

A total of 311 (32.81 %) students signed the informed consent and returned the completed questionnaire. They are young (mean age: 22.78 ± 3.80), and mostly female (*n* = 217). Less than 50 % (*n* = 149) of respondents are aware of the thirtieth anniversary of the Basaglia law [18]; about 80 % (*n* = 248) never participated in seminars or conferences on stigma, and only 33 % (*n* = 102) usually read newspapers for about 20 min every day (Table 1).

**Table 1:** Socio-demographic characteristics of the sample (*n* = 311)

Variable	Frequency (n)	Percentage (%)
Gender		
Male	94	30.23
Female	217	69.77
Civil status		
Single	143	45.98
Engaged	160	51.45
Married	7	2.25
Divorced	1	0.32
Knowledge about celebration of the thirtieth anniversary of the enactment of the Basaglia law?		
Yes	149	47.91
No	162	52.09
Participation in seminars or conferences related to the issue of stigma in psychiatry		
Yes	63	20.26
No	248	79.74
Daily time spent reading a newspaper (in minute)		
0	85	27.33
10	82	26.37
20	102	32.79
30	28	9.00
40	5	1.61
>40	9	2.89

### Confirmatory factor analysis

**Fig. 1:**
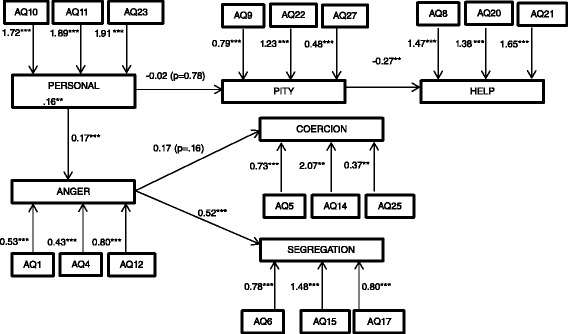
The six-factor measurement of the responsibility model (*** = *p* < 0.001; ** = *p* < 0.01; * = *p* < 0.05)

The path analysis of the attribution theory of personal responsibility for mental illness is shown in Fig. 1 [3]. The 18 items were defined as loading on six different first order latent factors [1, 30]: personal responsibility (10, 11, 23), pity (9, 22,27), help (8, 20, 21), anger (1, 4, 12), coercion (5, 14, 25) and segregation (6, 15, 17). The path analysis indicates the significance of the chi square value (*χ*2 = 308.53; df = 130; *p* < 0.01) and a value for the *χ*2/df ratio (2.37) above the reference value (2.0), which do not support fit. The GFI of 0.90 represents a good value, while both the AGFI (=0.86) and the CFI (=0.83) are slightly below the threshold. On the contrary, the value of the RMSEA (=0.07) is slightly above the recommended values. The covariance estimate among factors is significant, with the exception of the associations of “personal responsibility” with “pity” (-0.02; *p* = 0.78), and of “anger” with “coercion” (0.17; *p* = 0.16). All the items loaded significantly into their corresponding factors.

**Fig. 2:**
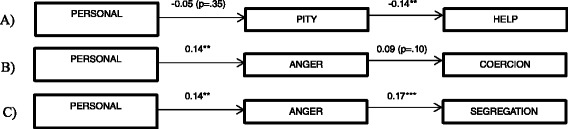
Sub-models **a**, **b** and **c** of the responsibility model (*** = *p* < 0.001; ** = *p* < 0.01; * = *p* < 0.05)

On the basis of the results provided above, the personal responsibility model was divided into three sub-models (Fig. 2). The path analysis of sub-model A (personal responsibility → pity → help) resulted in a significant chi square (*χ*2 = 66.18; df = 24; *p* < 0.001) and a *χ*2/df ratio (2.76) above the reference value (2.0), not supporting the fit. The other three fit indices indicated an acceptable model fit (GFI = 0.95; AGFI = 0.91; CFI = 0.91; RMSEA = 0.08). The second path consisted of sub-model B (personal responsibility → anger → coercion), and the path analysis resulted in a significant chi square (*χ*2 = 47.69; df = 24; *p* < 0.005) and a *χ*2/df ratio (1.98) below the reference value (2.0), thus supporting the fit. Two of the four other fit indices indicated an acceptable model fit (GFI = 0.97; AGFI = 0.94; CFI = 0.88; RMSEA = 0.06). The third path consisted of sub-model C (personal responsibility → anger → segregation), and the path analysis resulted in a significant chi square (*χ*2 = 185.63; df = 24; *p* < 0.005) and a *χ*2/df ratio (7.73) above the reference value (2.0), not supporting the fit. Only one of the four other fit indices indicated a good fit with the model (GFI = 0.91; AGFI = 0.87; CFI = 0.70; RMSEA = 0.09).

**Fig. 3:**
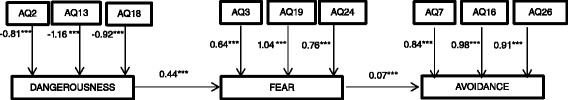
The three-factor measurement of the dangerousness model (*** = *p* < 0.001; ** = *p* < 0.01; * = *p* < 0.05)

The pathway analysis related to the danger appraisal towards persons with mental disorders is shown in Fig. 3 [3]. Nine items loaded into three different first order latent factors: dangerousness (items 2,13,18), fear (items 3,19,24) and avoidance (items 7,16,26). The pathway analysis showed a significant chi square value (*χ*2 = 61.96; df = 24; *p* < 0.01) and a value for *χ*2/df ratio (2.58) above the reference (2.0). The GFI (=0.96), AGFI (=0.93) and CFI (=0.96) showed very good values, while RMSEA = 0.07 was slightly above the recommended values. All items loaded significantly into the corresponding factors and the estimated covariance between factors was always significant, ranging from -1.16 to 1.04.

### Internal consistency reliability

Cronbach’s alpha, used to test internal consistency, was 0.68. The “Fear” subscale showed the highest value of Internal Consistency Reliability (0.83). Two other subscales (“Pity” = 0.63 and “Dangerousness” = 0.60) achieved a score slightly below the cut-off, while the “Responsibility” subscale obtained a very low value (0.48) (Table 2).

**Table 2:** Internal consistency (Cronbach’s alpha coefficient)

AQ-27-I	Internal consistency reliability (Cronbach’s alpha coefficient)
Responsibility	0.48
Pity	0.63
Anger	0.74
Dangerousness	0.60
Fear	0.83
Help	0.77
Coercion	0.63
Segregation	0.82
Avoidance	0.71
Total	0.68

### Analysis of dependence of AQ-27-I scores

Multiple linear regression analysis was used to develop a model for predicting the total score of the AQ-27-I and the score of the subscales using five independent variables (age, sex, time spent reading newspapers, knowledge of the Basaglia law and participation in conferences on stigma) (Table 3). Two subscales (responsibility and anger) did not show any association with the independent variables. The subscale avoidance was negatively associated with the independent variables “knowledge of the Basaglia law” (β = -0.18; *p* = 0.002) and “participation at conferences on stigma” (β = -0.13; *p* = 0.02). The independent variable “age” was negatively associated with four subscales of the AQ-27-I: coercion (β = -0.12; *p* = 0.04), segregation (β = -0.33; *p* = 0.001), fear (β = -0.12; *p* = 0.03) and avoidance (β = -0.11; *p* = 0.05). Two positive associations were found between the variable “time spent in reading newspapers daily” and the subscales segregation (β = 0.12; *p* = 0.03) and dangerousness (β = 0.11; *p* = 0.04). The independent variable “sex” was also related to the help (β = 0.16; *p* = 0.004) and dangerousness (β = -0.12; *p* = 0.04) subscales. Finally, the “Pity” subscale was associated with the variable “knowledge of the Basaglia law” (β = -0.12; *p* = 0.03). Using the total score of AQ-27-I as dependent variable only two hypothetic predictors were found: age (β = -0.12; *p* = 0.04) and time spent in reading newspapers (β = 0.17; *p* = 0.04)

**Table 3:** Multiple linear regression between the subscale and the total score of the AQ-27-I (dependent variables) and the other independent variables

	Age	Sex	Knowledge of Basaglia law	Conferences related to stigma	Daily time spent reading a newspaper
Responsibility	β = 0.03; *p* = 0.64	β = -0.05; *p* = 0.38	β = -0.05; *p* = 0.38	β = 0.04; *p* = 0.49	β = 0.11; *p* = 0.06
Pity	β = -0.01; *p* = 0.83	β = -0.10; *p* = 0.07	β = -0.12; *p* = 0.03	β = -0.01; *p* = 0.88	β = -0.02; *p* = 0.79
Help	β = -0.04; *p* = 0.46	β = 0.16; *p* = 0.004	β = 0.09; *p* = 0.11	β = 0.07; *p* = 0.21	β = 0.10; *p* = 0.09
Anger	β = 0.001; *p* = 0.98	β = -0.10; *p* = 0.07	β = 0.03; *p* = 0.56	β = 0.04; *p* = 0.51	β = 0.11; *p* = 0.06
Coercion	β = -0.12; *p* = 0.04	β = 0.04; *p* = 0.40	β = 0.04; *p* = 0.56	β = 0.10; *p* = 0.08	β = 0.01; *p* = 0.79
Segregation	β = -0.33; *p* = 0.001	β = -0.08; *p* = 0.16	β = -0.11; *p* = 0.06	β = -0.09; *p* = 0.10	β = 0.12; *p* = 0.03
Dangerousness	β = -0.003; *p* = 0.96	β = -0.12; *p* = 0.04	β = 0.06; *p* = 0.92	β = 0.02; *p* = 0.69	β = 0.11; *p* = 0.04
Fear	β = -0.12; *p* = 0.03	β = 0.04; *p* = 0.49	β = -0.07; *p* = 0.23	β = -0.02; *p* = 0.66	β = 0.07; *p* = 0.20
Avoidance	β = -0.11; *p* = 0.05	β = -0.08; *p* = 0.16	β = -0.18; *p* = 0.002	β = -0.13; *p* = 0.02	β = -0.08; *p* = 0.16
Total Score	β = -0.12; *p* = 0.04	β = -0.05; *p* = 0.45	β = -0.08; *p* = 0.32	β = 0.07; *p* = 0.30	β = 0.17; *p* = 0.04

## Discussion

The primary aim of the study was to validate the AQ-27-I in a medical student population. The confirmatory factor analysis of the model of personal responsibility scored slightly below the reference ranges (with the exception of the GFI, which was 0.90). The results of the path analysis were only partially overlapping with the previous validation: in particular, we did not find a significant association between anger and coercion, and between pity and help, and the *χ*2/df ratio did not support the fit. The behavioral response of segregation may be considered more appropriate to the emotional state of anger due to the fact that the individual with mental illness is considered responsible for his problems [31-34].

The results obtained from the second model (“Danger Appraisal model”) are more robust, but again the *χ*2/df ratio does not support the fit. All covariances between the various factors of the model have reached a statistical significance (“dangerousness” with “fear” and “fear” with “avoidance”). Additionally, all the nine items loaded in their respective three factors. With regard to the fit indices, the results were satisfactory: only the RMSEA was above the cut-off, while all other indicators (GFI, AGFI and CFI) were within the cut-off. Therefore, the danger appraisal model seems to be a better fit with the Italian cultural context and it is more applicable in medical students. The internal consistency reliability of the questionnaire is 0.68. Three factors (dangerousness, fear and coercion) were below the threshold. This result suggests that other confounding variables could play an important role in the remaining variance.

The second aim of this study was to define possible predictors of stigmatizing attitudes in a medical student population. The analysis of associations between the subscales and the total score of AQ-27-I with the other collected variables for defining possible stigmatizing attitudes in a university student population has allowed us to confirm data already available in the literature. In particular, our results show that being female is associated with a lower score on the dangerousness subscale and a higher score on the help subscale, thus confirming that negative prejudices against people with psychiatric disorders are higher in males [35].

Another interesting finding is the inversely proportional association between age as independent variable and the subscales coercion, segregation and dangerousness: the subscales’ scores decrease as age increases. We can assume that during their degree course, students address issues related to ethics, deontology and the relationship between health professionals and patients, thus reducing their stigmatizing behaviors and stereotypes.

One of the main sources of stigma is how people with mental disorders are portrayed in newspapers: unreliable, insecure and dangerous [36, 37]. In our study, this hypothesis is strengthened by the direct relationship between time spent in reading newspapers and the sense of danger, with the consequent desire of segregation, caused by people with psychiatric disorders.

The attendance in events on stigma is associated with less avoidance. However, from the evidence available in the literature [38] about the effectiveness of educational interventions as a mean to fight stigma, this finding requires more confirmation and it has to be considered speculative.

Finally, it is interesting to note that the knowledge of the thirtieth anniversary of the Basaglia law is associated with lower levels of stigmatizing attitudes (i.e., low scores on the pity and avoidance subscales) [39]. Although this association can be by chance, it is likely that the principles of the Italian law have been well received by the population, and that patients with mental disorders are not avoided.

## Conclusions

This cross-sectional study has some limitations, which have to be considered. The main limitation is that unfortunately we did not consider the strongest predictor of positive attitudes, which is knowing someone with a mental health problem (social contact) [17, 40]. Second, the sample included medical students who have agreed to participate in the survey because of their interest in the topic, and we do not have information on students who did not agree to participate. Third, participants were recruited at the Faculty of Medicine, and it may be that these students are more sensitive on social issues. Further studies should explore this phenomenon in students from other areas of knowledge. Fourth, we had a quite low response rate (32.8 %; 311/948), which may be due to lack of time to fill in the questionnaire or to have not received the invitation by e-mail. Although the sample size was sufficient enough to carry out the study and to perform statistical analyses, we acknowledge that the response rate might have biased the results, since those who responded may be more interested in the topic.

The analysis of possible characteristics associated with AQ-27-I subscales represents a first step for explaining the dynamics of stigma among university students: however, in order to have a clearer picture of this phenomenon, it is necessary to proceed with the definition of more complex models. Our findings confirm the need to improve strategies to fight stigma in university contexts. In particular, it may be relevant to include different teaching activities, such as seminars, first-person accounts and educational materials on stigma, or to involve mass media [41]. With these activities, we could verify which strategy (educational or contact) is most effective for reducing stigma and discrimination in school populations.
